# Exogenous stromal cell-derived factor-1 (SDF-1) suppresses the NLRP3 inflammasome and inhibits pyroptosis in synoviocytes from osteoarthritic joints via activation of the AMPK signaling pathway

**DOI:** 10.1007/s10787-021-00814-x

**Published:** 2021-06-03

**Authors:** Shuya Wang, Ali Mobasheri, Yue Zhang, Yanli Wang, Tianqi Dai, Zhiyi Zhang

**Affiliations:** 1 Department of Rheumatology, The First Affiliated Hospital of Harbin Medical University, No. 23 Youzheng St, Harbin, 150001 China; 2 Department of Joint Surgery, First Affiliated Hospital of Sun Yat-sen University, Guangzhou, 510080 Guangdong China; 3 Research Unit of Medical Imaging, Physics and Technology, Faculty of Medicine, University of Oulu, PO Box 5000, 90014 Oulu, Finland; 4 Department of Regenerative Medicine, State Research Institute Centre for Innovative Medicine, 08406 Vilnius, Lithuania; 5 Department of Orthopedics, Rheumatology and Clinical Immunology, University Medical Center Utrecht, 508 GA Utrecht, The Netherlands

**Keywords:** Osteoarthritis (OA), Low-grade inflammation, Stromal cell-derived factor-1, NLRP3 inflammasome, Pyroptosis, Synoviocyte, Synovitis

## Abstract

**Objective:**

NLRP3 inflammasome may play a key role in OA pathogenesis. Stromal cell-derived factor-1 (SDF-1) is a homeostatic CXC chemokine. Since the role of SDF-1 in OA has not been explored, this study aimed to examine the effect of SDF-1 on NLRP3 inflammasome and pyroptosis in synoviocytes from OA joints.

**Materials and methods:**

Human synovium was obtained from OA patients for isolation of primary synoviocytes and a murine model of collagenase-induced OA was established for testing intra-articular injections of SDF-1. Immunoblotting assays were used to examine the effects and underlying mechanism of action of SDF-1 on NLRP3 inflammasome and synoviocyte pyroptosis in synoviocytes. Inhibitors of AMPK and PI3K–mTOR were utilized to investigate the key signaling pathways involved in SDF-1-mediated OA inflammasome formation and pyroptosis.

**Results:**

Synoviocytes from OA joints exhibited significantly higher expression of NLRP3 inflammasome and biomarkers of synoviocyte pyroptosis relative to healthy individuals. This was confirmed in the collagenase-induced OA model, where OA synoviocytes had a significantly lower SDF-1 expression than healthy ones. SDF-1 treatment in synoviocytes of OA patients and collagenase-induced OA led to significant downregulation in the expression of NLRP3 inflammasome and synoviocyte pyroptosis biomarkers. Inhibition of the AMPK signaling pathway significantly suppressed the inhibitory effect of SDF-1 on NLRP3 inflammasome expression of OA synoviocytes. However, blocking the SDF-1-activated PI3K–mTOR signaling pathway could still suppress the expression of NLRP3 inflammasome and synoviocyte pyroptosis biomarkers.

**Conclusions:**

SDF-1 ameliorates NLRP3 inflammasome and pyroptosis in OA synoviocytes through activation of the AMPK signaling pathway. Therefore, SDF-1 may be a novel therapeutic target for OA.

**Supplementary Information:**

The online version contains supplementary material available at 10.1007/s10787-021-00814-x.

## Introduction

Osteoarthritis (OA) is the most common form of arthritis and a highly prevalent and disabling condition that affects movable joints, such as knees, hips, and hands (Katz et al. 2021). Currently, OA is considered to be a whole joint disease, as opposed to a simple cartilage wear and tear disease (Deveza and Loeser 2018). The pathogenesis of OA involves mechanical (Hunt et al. 2020), metabolic (Zheng et al. 2021), and inflammatory factors (Hunter and Bierma-Zeinstra 2019; Chow and Chin 2020). Recent research suggests that low-grade inflammation plays an important role in the development and progression of OA (Scanzello 2017). There are no effective disease-modifying osteoarthritis drugs (DMOADs) which have been approved by the regulatory agency (Oo et al. 2018); therefore, we urgently need to find new targets for the development of effective therapeutics.

Stromal cell-derived factor-1 (SDF-1), also known as CXCL12, is a member of the homeostatic CXC family of chemokines, with emerging roles in bones, joints, and muscles (Gilbert et al. 2019). However, the role of SDF-1 in the pathogenesis of OA has yet to be determined. Recent research has showed that SDF-1 levels increase significantly in OA synovial fluid (Kanbe et al. 2002). Previous studies have speculated that SDF-1 may play a protective role in OA by inducing significant extents of osteoblast proliferation and upregulating the expression of collagen type I in OA patients (Lisignoli et al. 2006; Shen et al. 2014). We have previously reported that SDF-1 can increase vascular endothelial growth factor expression in chondrogenic progenitor cells (Wang et al. 2017). However, thus far, no study has been conducted to examine the effect of SDF-1 on the NLRP3 inflammasome and pyroptosis in OA or explored its underlying mechanism of action.

The inflammasome can promote inflammation by activating the cysteinyl aspartate specific proteinase (caspase-1) protease, a pro-inflammatory caspase and a key regulator of cell death (Martinon and Tschopp 2007). The NLRP3 inflammasome is known to be activated in a two-step process (Tschopp and Schroder 2010; Jo et al. 2016). First, toll-like receptors recognize endogenous material or microbial materials and trigger increased expression of pro-interleukin-(IL)1β, pro-IL-18, and NLRP3 (Sandanger et al. 2013; Kelley et al. 2019). In the second step, an activation signal initiates NLRP3 oligomerization, which further recruits NLRP3, adapter protein apoptosis-associated speck-like protein (ASC), and pro-caspase-1 to form the inflammasome (Kelley et al. 2019). These processes result in the conversion of pro-caspase-1 to caspase-1, which, in turn, proteolytically cleaves pro-IL-18 and pro-IL-1β and subsequently triggering their activation and release (Franchi et al. 2009). Finally, this step induces pyroptosis through cleavage of gasdermin D (GSDMD) (Shi et al. 2017). A recent study reported the involvement of the NLRP3 inflammasome in the pathogenesis of OA (McAllister et al. 2018). In addition, IL-1β synthesized by synoviocytes from the inflamed synovium have been shown to exacerbate the pro-degradative mechanisms responsible for cartilage breakdown (Sellam and Berenbaum 2010). This highlights the importance of the synovium in driving inflammatory processes within the joint in OA (Scanzello and Goldring 2012; Liu-Bryan 2013).

OA is a complex and serious disease (Hawker 2019) and there are no effective drugs available for its treatment. The NLRP3 inflammasome plays a unique role in the pathogenesis of synovitis (Jin et al. 2011), which is an important mechanism underlying OA pathogenesis (Scanzello and Goldring 2012). To the best of our knowledge, no experimental study has yet examined the effects of SDF-1 on the NLRP3 inflammasome and pyroptosis in OA. We hypothesized that SDF-1 can decrease expression of the NLRP3 inflammasome and related proteins in synoviocytes from OA joints exhibiting synovitis and may have potential as a novel therapeutic target for synovitis in OA. Therefore, we utilized recombinant SDF-1 to treat fibroblast-like synoviocytes (FLS) from human OA joints and synoviocytes derived from collagenase-induced OA mice and explored the effects and underlying mechanism of action of SDF-1 in these in vitro and preclinical models of inflammatory OA.

## Materials and methods

### Materials

SDF-1 (purity is > 97%, by SDS-PAGE under reducing conditions and visualized by silver stain) was purchased from R&D Systems. Dorsomorphin (Compound C, CC), inhibitor of phosphatidylinositol 3-kinase (PI3K)-mammalian target of rapamycin (mTOR), and 3-methyladenine (3MA) were purchased from MedChemExpress. Anti-NLRP3, anti-caspase-1, anti-IL-1β, and anti-PI3K antibodies were purchased from Abcam. Anti-ASC antibody was purchased from Santa Cruz Biotechnology. Anti-GSDMD and anti-microtubule-associated protein light chain 3 (LC3) antibodies were purchased from Cell Signaling Technology. Anti-phospho-adenosine 5′-monophosphate-activated protein kinase (AMPK) α1/2, anti-GAPDH, horseradish peroxidase (HRP) goat anti-rabbit IgG, and HRP goat anti-mouse IgG antibodies were purchased from Abbkine. Anti-mTOR antibody was purchased from Bioworld Technology. Anti-p62 antibody was purchased from ABclonal.

### In vitro culture of healthy and OA FLS and SDF-1 stimulation of OA FLS

Healthy primary FLS were purchased from Procell. Synovium was isolated from the knee of OA patients at the time of all knee replacement surgery carried out in the Department of Orthopaedics, the First Affiliated Hospital of Harbin Medical University. Knee OA was diagnosed according to the criteria established by the American College of Rheumatology (Altman et al. 1986). Informed consent was obtained from all patients participating in the study. Synovial tissues were washed three times with phosphate-buffered saline (PBS), cut into sections with an area of 2–3 mm^2^, and digested in 2 mg/ml collagenase type II (Sigma-Aldrich) at 37 ℃ overnight and isolated cells were filtered through a cell strainer (Alvaro-Gracia et al. 1990). Healthy human FLS and OA-derived FLS were used between 4 and 6 passages. OA FLS were treated with SDF-1 (20, 50, or 100 ng/ml) for 24 h, or SDF-1 (100 ng/ml) for 1, 3, 6, 12, or 24 h. To identify the signaling pathways involved in the effect of SDF-1, OA-derived FLS were pretreated with the AMPK inhibitor CC (10 μmol/ml) for 1 h or the PI3K–mTOR inhibitor 3MA (5 mM) for 2 h prior to the administration of SDF-1 (100 ng/ml).

### In vitro proliferation assay

Proliferation of OA-derived FLS was evaluated using the Cell Counting Kit-8 (CCK-8, Dojindo Molecular Technologies), as described previously (Wang et al. 2019). Briefly, 10^4^ cells were seeded in a 96-well plate. After 48 h, these cells were treated with different concentrations of SDF-1 added to fresh medium and incubated at 37 ℃ for 24 h. The plates were then read at a wavelength of 450 nm. The experiment was repeated three times.

### Gene expression analysis

Total RNA was extracted from OA-derived FLS with the TRIzol reagent (Invitrogen) and reverse-transcribed to cDNA. Real-time PCR analysis was carried out using TOYOBO reverse transcription reagents and SYBR Green PCR Master Mix (Bio-Rad). Specific primers were custom synthesized from Ruibiotech (Supplementary Table 1). *GAPDH* was used as a reference gene. All samples were measured in triplicates and the relative quantification method ($2^{{ - \Delta \Delta C_{{\text{T}}} }}$) was used to calculate the relative expression levels of genes in the different groups (Wang et al. 2017).

### Western blotting

Protein expression levels in OA-derived FLS were estimated by western blotting, as previously described with some modifications (Zhang et al. 2015). Total protein from healthy or OA FLS cultures was extracted with cold RIPA lysis buffer and Bio-Rad assayed. Extracted proteins were then separated using 12.5 or 7.5% sodium dodecyl sulfate–polyacrylamide gel electrophoresis (SDS-PAGE) and transferred to polyvinylidene fluoride (PVDF) membranes. The blots were probed with anti-NLRP3 (1:1000), anti-ASC (1:800), anti-caspase-1 (1:1000), anti-GSDMD (1:1000), anti-IL-1β (1:1000), anti-phosphorylated-AMPK (1:1000), anti-phosphorylated-PI3K (1:1000), anti-mTOR (1:1000), anti-LC3 (1:1000), anti-p62 (1:1000), and anti-GAPDH (1:5000) antibodies at 4 ℃ overnight, followed by incubation with an HRP-conjugated goat anti-rabbit or goat anti-mouse IgG secondary antibody (1:10,000) for 2 h at room temperature. The labeled blots were finally imaged and analyzed using the ImageJ software (National Institutes of Health, Bethesda, USA).

### Establishment of collagenase-induced OA model and SDF-1 treatment

Twelve-week-old male C57BL/6 mice were obtained from the Second Affiliated Hospital of Harbin Medical University Animal Centre (Harbin, China) and maintained at the Laboratory Animal Centre, the First Affiliated Hospital of Harbin Medical University. Mice were housed with free access to water and food. Animal experiments were performed according to the guidelines of the National Institutes of Health for the Care and Use of Laboratory Animals (Zhang et al. 2015). The study was conducted in accordance with the Declaration of Helsinki and was approved by the Ethics Committee of the First Affiliated Hospital of Harbin Medical University (IRB: 2018139). A collagenase-induced OA mouse model was established as previously described, with minor modifications (van der Kraan et al. 1990). Briefly, OA was induced in mice using two intra-articular injections of 5 U collagenase type VII (Sigma-Aldrich) on day 0 and day 2 in the right knee. Administration of collagenase type VII is known to damage the cruciate and collateral ligaments, leading to instability of knee joints, and finally resulting in chronic synovial activation and cartilage destruction, which represented an OA-like phenotype. Then, SDF-1 (120 ng/kg) was injected twice a week in the knee joint of collagenase-induced OA mice from day 7. Day 42 represented the end point of the establishment of the disease model. The control group consisted of knee joints injected with saline (*n* = 3 for each group).

### Microcomputed tomography (micro-CT) imaging

On day 42 following the initial collagenase VII injection, mice were killed. The distal femur and proximal tibia were cut with blunt scissors to obtain knee joint tissues and scanned with a Quantum GX microcomputed tomography (micro-CT) imaging system (Perkin Elmer, Waltham, MA, USA) (Wang et al. 2019) to investigate the effect of SDF-1 on the three-dimensional (3D) bone structure in collagenase-induced OA mice. The voxel size was 20 μm, X-ray tube voltage was 70 kV, current was set to 88 μA, and the exposure time was 14 min. The acquired images were reconstructed into a 3D representation to exhibit structural changes induced by OA.

### Haematoxylin and eosin (H&E) staining

Whole knee joints were collected and fixed in 4% formalin, decalcified in 4% formic acid in PBS, and then decalcified in 10% ethylenediaminetetraacetic acid (Sigma-Aldrich, Shanghai, China) for 4 weeks. The tissue was dehydrated in a solvent system containing increasing amount of alcohol, and then embedded in paraffin. Coronal sections (5-μm thickness) were cut and stained with hematoxylin and eosin (H&E). Two independent observers scored the histological changes based on synovial hyperplasia, joint inflammation, and bone erosion (Wang et al. 2019).

### Immunohistochemistry (IHC)

Immunostaining of knee joint sections was performed with specific antibodies against target proteins according to a previously published protocol with some modifications (Wang et al. 2019). Sections were incubated with anti-NLRP3 (1:100), anti-ASC (1:100), anti-caspase-1 (Wanleibio, 1:100), anti-GSDMD (Affinity Biosciences, 1:100), and anti-IL-1β (1:100) antibodies overnight at 4 °C. On the following day, these tissue sections were incubated with a polymer-HRP detection system (PV9001, ZSGB-BIO) and visualized with a diaminobenzidine (DAB) peroxidase substrate kit (ZLI-9017, ZSGB-BIO). Each section was evaluated under a microscope (DMi8, LEICA) at 20 × magnification. Histological analyses were performed in a blinded manner by two independent observers. Image-ProPlus6 software 6.0 (Media Cybernetics, Inc.) was used to analyze the average integrated optical density (IOD) of three randomly selected areas of the acquired images.

### Statistical analysis

All data collected are represented as mean ± standard error of the mean (SEM). GraphPad Prism 5.0 software (San Diego, CA, USA) was used for statistical analysis. Data were checked for statistical significance using Student’s *t*-test or one-way analysis of variance (ANOVA). Values of *p* < 0.05 were considered to indicate a statistically significant difference.

## Results

### SDF-1 suppressed NLRP3 inflammasome and pyroptosis of OA FLS

**Fig. 1:**
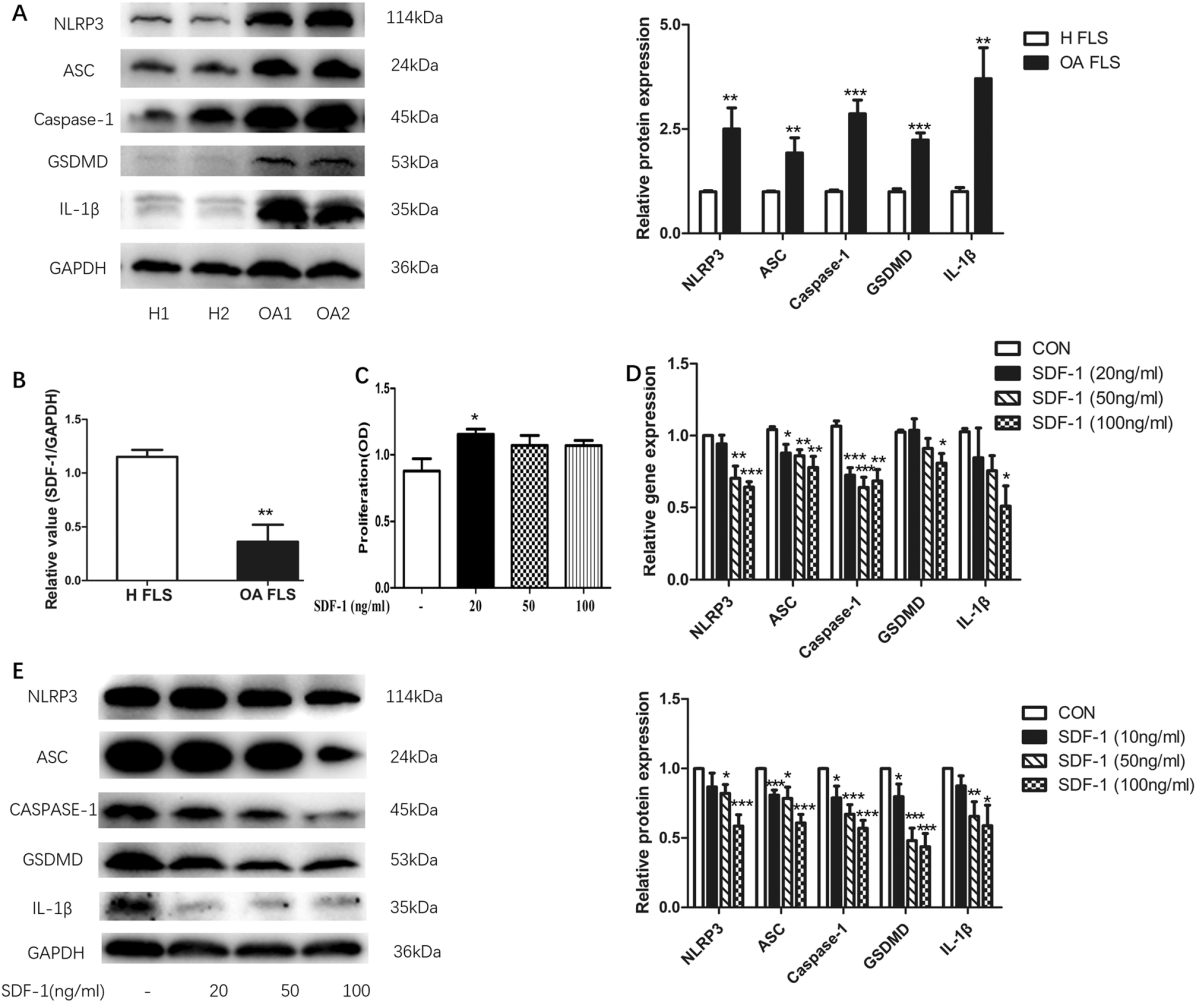
SDF-1 suppressed NLRP3 inflammasome and pyroptosis of OA FLS. NLRP3, ASC, caspase-1, GSDMD and IL-1β protein expression in healthy FLS and OA FLS were estimated by western blotting (**a**, *n* = 9). Data are expressed as means ± standard error of the mean (SEM). In addition, SDF-1 gene expressions in healthy FLS and OA FLS were detected by RT-PCR (**b**, *n* = 5). After treatment with different concentrations of SDF-1 (20, 50, and 100 ng/ml) for 24 h (*n* = 6), CCK-8 was added to OA FLS and incubated for 1 h. Absorbance (OD, optical density value) at 450 nm was detected by a BioTek microplate reader (**c**) (OD, optical density value). SDF-1 downregulated the gene (**d**) and protein (**e**) expression levels of NLRP3 inflammasome, GSDMD, and IL-1β, which represented pyroptosis (*p* < 0.05, *n* = 6–8). *p < 0.05; **p < 0.01; ***p < 0.001 versus control; CON, control: OA FLS treated only with the medium

OA-derived FLS exhibited a significantly higher expression of the NLRP3 inflammasome and pyroptosis-related proteins, GSDMD and IL-1β, compared to healthy synoviocytes (*p* < 0.05). The expression levels of NLRP3 inflammasome (NLRP3, ASC, and caspase-1), GSDMD, and IL-1β were significantly upregulated in OA FLS compared with that in healthy FLS (*p* < 0.05, *n* = 9), as estimated by western blotting (Fig. 1a). OA FLS also had a significantly lower gene expression of SDF-1 (*p* < 0.05, *n* = 5) compared to healthy FLS (Fig. 1b).

To further study the effect of SDF-1 on OA FLS inflammasome and pyroptosis, a series of experiments were carried out. First, to rule out any cytotoxic effects, we examined the influence, if any, of SDF-1 on the proliferation of OA FLS. Only SDF-1 at a concentration of 20 ng/ml was found to increase the proliferation of OA FLS (*p* < 0.05, *n* = 6); the other two concentrations of SDF-1 used in these experiments had no significant influence on cell proliferation (*p* > 0.05) (Fig. 1c). OA FLS were examined after treatment with different doses of SDF-1 (20, 50, and 100 ng/ml). Gene (Fig. 1d) and protein (Fig. 1e) expression levels of the NLRP3 inflammasome (NLRP3, ASC, and caspase-1), GSDMD, and IL-1β were significantly downregulated on SDF-1 treatment, with the maximal effect observed at a 100 ng/ml concentration (*p* < 0.05, *n* = 6–8).

### SDF-1 decreased NLRP3 inflammasome and pyroptosis in collagenase-induced OA mouse model

**Fig. 2:**
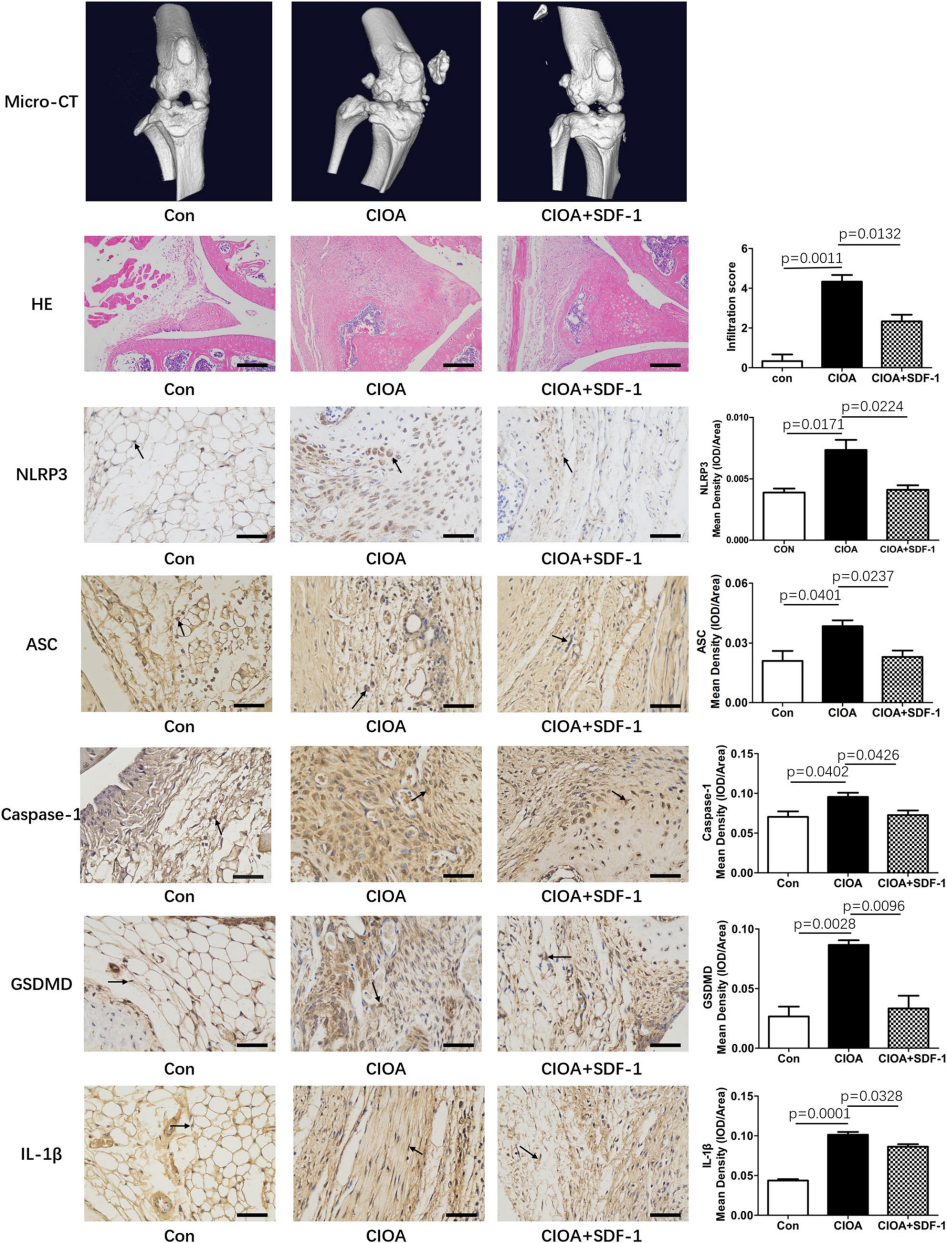
SDF-1 decreased NLRP3 inflammasome in collagenase-induced OA mouse model. Type VII collagenase injection can cause narrowing of the joint space and osteophyte formation in Micro-CT and H&E staining. However, intra-articular injection of 120 ng/kg SDF-1 twice a week delayed the pathological progression of these symptoms. IHC results showed that the synovium of collagenase-induced OA mice had a significantly higher expression of NLRP3, ASC, caspase-1, and protein markers of pyroptosis (GSDMD and IL-1β) compared to the synovium of control mice (*p* < 0.05) (*n* = 3); however, SDF-1 treatment could reverse this effect to some extent. IOD, integrated optical density

To examine the effect of SDF-1 in vivo, we treated collagenase-induced OA mice by intra-articular injection of SDF-1 (recombinant human SDF-1 was reconstituted in sterile PBS containing at least 0.1% bovine serum albumin, and the treatment dosage of SDF-1 used was 240 ng/kg per week). Micro-CT results showed that at week 7 of the treatment, collagenase-induced OA mice exhibited narrowing joint space and osteophyte formation. Alongside type VII collagenase injection for a week, further SDF-1 treatment was observed to delay these pathological phenotypes. These results could also be confirmed from inspection of the H&E stained tissue sections. Furthermore, IHC staining also showed that collagenase-induced OA mice had a significantly higher expression of NLRP3, ASC, caspase-1, GSDMD, and IL-1β than mice in the control group (*p* < 0.05). Conversely, SDF-1 treatment decreased their protein level expression (*p* < 0.05) (Fig. 2).

### SDF-1 suppressed NLRP3 inflammasome expression and pyroptosis of OA FLS through AMPK signaling pathway

**Fig. 3:**
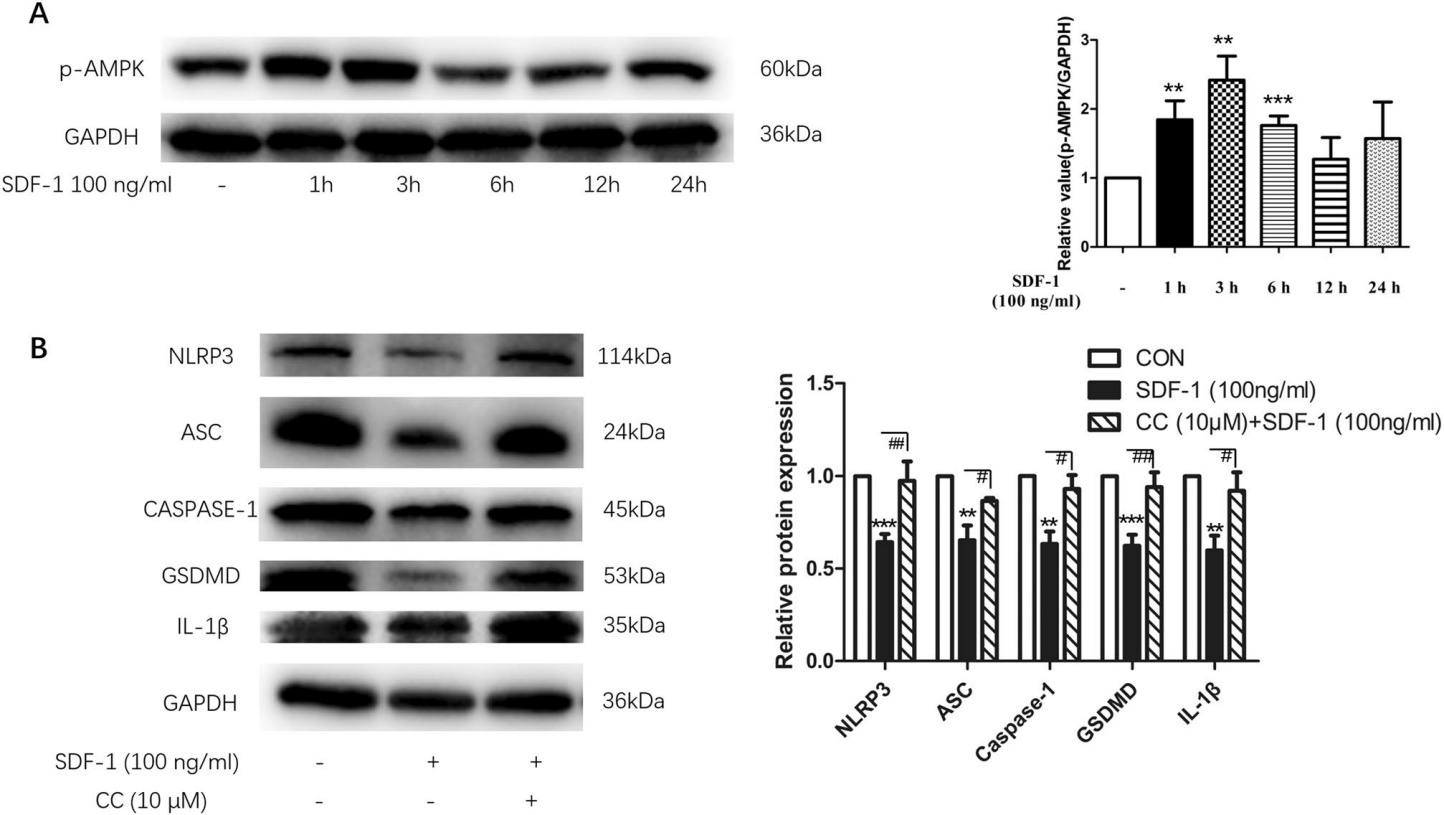
SDF-1 suppressed expression of NLRP3 inflammasome and pyroptosis in OA FLS through AMPK signaling pathway. SDF-1 was found to activate the AMPK signaling pathway at 1-, 3-, and 6-h time points, with the maximal change observed at 3 h (**a**) (*n* = 8). Pre-treatment of OA FLS with 10 μM CC (an AMPK signaling pathway inhibitor) for 1 h led to suppression of the inhibitory effect of SDF-1 on NLRP3 inflammasome and pyroptosis (**b**) (*n* = 6). *p < 0.05; **p < 0.01; ***p < 0.001 versus control; CON, control: OA FLS treated with only the medium; #*p* < 0.05; ##*p* < 0.01; ###*p* < 0.001 versus SDF-1 (100 ng/ml) treatment

Next, we explored how SDF-1 contributed to the inhibitory effect mentioned earlier. First, our results showed that SDF-1 (100 ng/ml) increased the expression of phosphorylated-AMPK (p-AMPK), indicating that SDF-1 activates the AMPK signaling pathway. This effect reached a peak at the 3 h time point after injection. After treatment with SDF-1 (100 ng/ml) for more than 6 h, the activity of the AMPK pathway decreased (Fig. 3a) (*p* < 0.05, *n* = 8). OA FLS were then treated with SDF-1 (100 ng/ml) for 24 h with/out pre-treatment with the inhibitor of AMPK signaling pathway CC (10 μM) for 1 h. CC treatment could reverse the inhibitory effect of SDF-1 on the expression of NLRP3 inflammasome in OA FLS (Fig. 3b) (*p* < 0.05, *n* = 6). This revealed that the AMPK signaling pathway plays an important role in the function of SDF-1.

### Role of PI3K–mTOR signaling pathway in SDF-1-mediated suppression of NLRP3 inflammasome expression in OA FLS

**Fig. 4:**
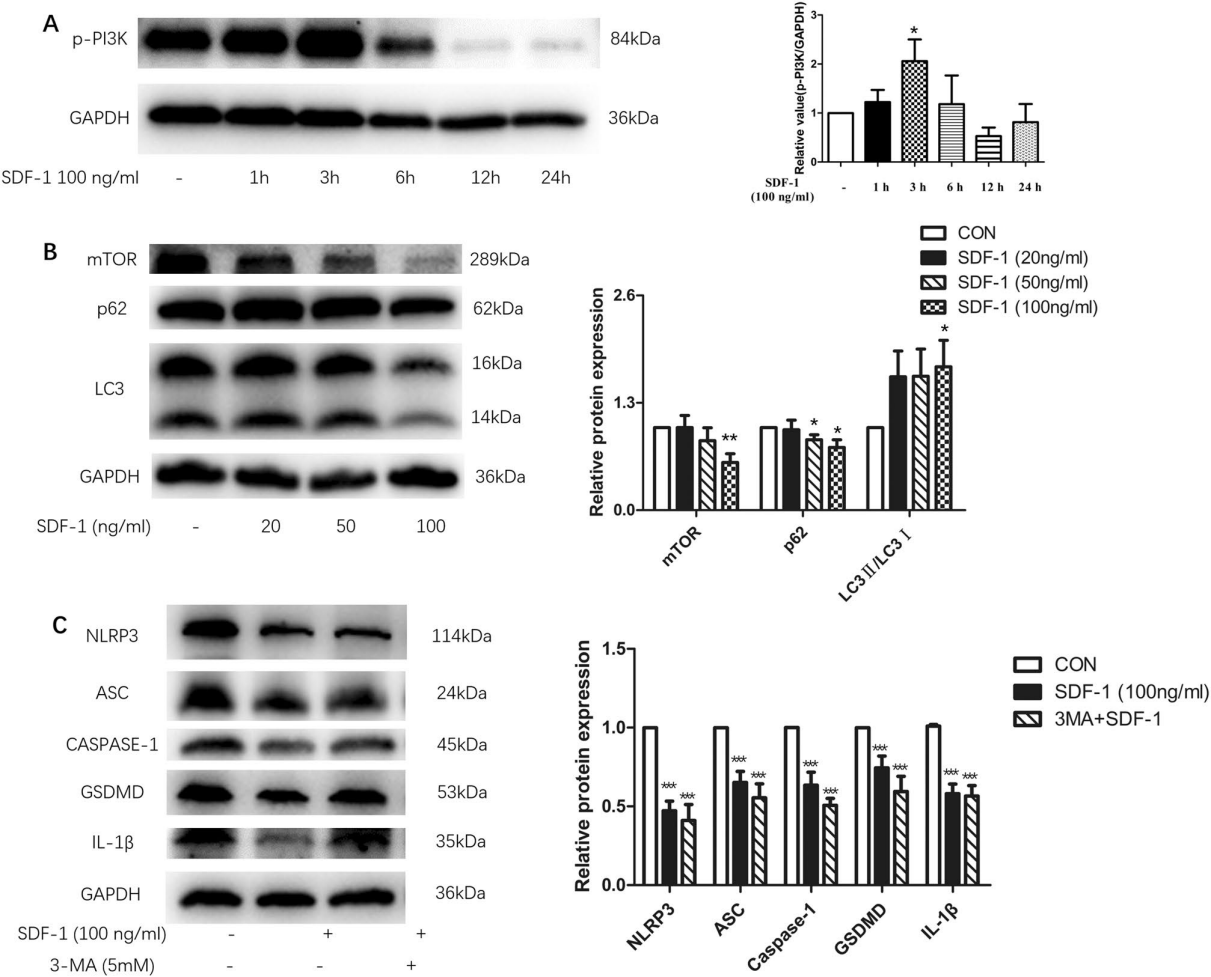
Downregulation of OA FLS inflammasome and pyroptosis by SDF-1 is not mediated by the PI3K–mTOR signaling pathway. SDF-1 activated the PI3K signaling pathway at 3 h (**a**) (*n* = 6). Besides, SDF-1 (100 ng/ml) decreased the protein expression of mTOR and p62 in OA FLS and increased the protein expression level of LC3-II/I (**b**) (*n* = 6–12). Pre-treated OA FLS with 5 mM 3MA (inhibitor of PI3K–mTOR) for 2 h could not reverse the inhibitory effects of SDF-1 (**c**) (*n* = 6). *p < 0.05;  **p < 0.01; ***p < 0.001 versus controls

Furthermore, to explore the contribution of the AMPK signaling pathway, we studied the influence of SDF-1 in the PI3K–mTOR signaling pathway. Our results showed that SDF-1 increased the protein levels of p-PI3K and LC3-II/I (Fig. 4a, b), and decreased protein levels of mTOR and p62 (Fig. 4b). The effect of SDF-1 on p-PI3K was also found to peak at 3 h. This means that SDF-1 activates both the PI3K–mTOR signaling and autophagic pathways. We also treated OA FLS with 3MA before SDF-1 treatment. Unexpectedly, inhibition of the PI3K–mTOR signaling pathway by 3MA (3-methyladenine, 3-MA, is a highly selective inhibitor of autophagy through PI3K signaling pathway) could not sufficiently reverse the inhibitory effect of SDF-1 on NLRP3 inflammasome and pyroptosis of OA synoviocytes (Fig. 4c).

## Discussion

OA is a complex chronic disease and synovitis plays a prominent role in OA pathogenesis (Han et al. 2020). The NLRP3 inflammasome is also known to be associated with synovitis (Clavijo-Cornejo et al. 2016). However, no previously published study has explored whether SDF-1 may exert an effect on NLRP3 inflammasome and pyroptosis in OA or investigated the underlying mechanism of action, if any. Our results suggest that OA synoviocytes had significantly higher expression of NLRP3 inflammasome biomarkers (NLRP3, ASC, and caspase-1) and pyroptosis-related proteins (GSDMD and IL-1β) compared to healthy synoviocytes. These results were consistent with those of a previous study (Clavijo-Cornejo et al. 2016). Meanwhile, we also observed that OA FLS was characterized by a significantly lower gene expression of SDF-1 compared to that in healthy FLS. We then explored if SDF-1 treatment could slow down OA pathogenesis, and indeed observed decreased expression of NLRP3 inflammasome biomarkers in the presence of SDF-1. Collectively, our observations indicate that SDF-1 may have exerted some beneficial effect on the collagenase-induced OA model. These functions of SDF-1 have been confirmed, in part, by another indirect study. Intrathecal application of SDF-1a could regulate NLRP3 inflammasome expression in spinal cord injury model (Zendedel et al. 2016).

To the best of our knowledge, this is the first report on the effect and underlying mechanism of action of SDF-1 on the expression of NLRP3 inflammasome in OA synoviocytes. Results from phosphospecific immunoblotting assay confirmed that SDF-1 could activate the AMPK and PI3K–mTOR signaling pathways. In addition, SDF-1-mediated downregulation of NLRP3 inflammasome, GSDMD, and IL-1β was substantially blocked only by the AMPK inhibitor (CC), but not by the PI3K–mTOR signaling pathway inhibitor (3MA). These results revealed that SDF-1 inhibits the OA FLS NLRP3 inflammasome and pyroptosis through the AMPK signaling pathway, while the PI3K–mTOR pathway counteracts the effect of SDF-1 (Supplementary Fig. 1). Based on these observations, we proposed a novel mechanism for SDF-1 in OA treatment. To the best of our knowledge, inhibitors of SDF-1 (such as AMD3100) have been previously reported to ameliorate degradation of OA cartilage (Lu et al. 2019). However, our study represents the first report confirming that treatment of human OA-derived primary synoviocytes with SDF-1 has these beneficial effects. This is relevant since differences in OA animal models or species-specific differences could account for variations in treatment efficacy in preclinical animal models and human patients. AMD3100 is known to be an inhibitor of the SDF-1 receptor CXC chemokine receptor 4 (CXCR4), while SDF-1 also binds to other receptors, such as CXCR7 and atypical chemokine receptor 3 (ACKR3) (Wang et al. 2018). Therefore, the effect of AMD3100 may not comprehensively represent the function of SDF-1 protein, especially in a clinical setting.

The limitations of the present study are as follows: first, we obtained synovial tissues from a limited number of patients with local OA; therefore, future studies will expand the sample size to generalize our findings to a much wider patient population. Second, these observations must be verified using alternative techniques, for example immunofluorescence and electron microscopy.

## Conclusion

In summary, our results demonstrate that key biomarkers and molecular components of the NLRP3 inflammasome and pyroptosis exhibit significantly higher expression in OA FLS compared to healthy FLS. Consistent with these observations, the synovium of collagenase-induced OA mice was also shown to express significantly higher levels of these proteins compared to healthy synovium from control mice. The NLRP3 inflammasome and pyroptosis may, therefore, influence the course of disease progression and adversely affect OA patients. SDF-1 treatment could inhibit the NLRP3 inflammasome and pyroptosis of OA FLS and synovium of collagenase-induced OA mice. Furthermore, our investigation of downstream signaling pathways revealed that SDF-1 is a putative cytoprotective protein that suppresses OA FLS NLRP3 inflammasome and pyroptosis through the AMPK signaling pathway, with the PI3K–mTOR signaling pathway counteracting the effect of SDF-1. These novel observations represent, to the best of our knowledge, the first report of insights into the effects and underlying mechanism of action of SDF-1-mediated inhibition of the OA NLRP3 inflammasome and pyroptosis. The data presented establish a new line of evidence to support the potential suitability of SDF-1 as a novel experimental therapeutic and cytoprotective agent for treating OA.

## Supplementary Information

Below is the link to the electronic supplementary material.

Supplementary file1 (DOCX 358 kb)

Supplementary file2 (DOCX 13 kb)

Supplementary file3 (PPTX 19610 kb)

Supplementary file4 (DOCX 14 kb)
