# Supplementary material for: Exogenous stromal cell-derived factor-1 (SDF-1) suppresses the NLRP3 inflammasome and inhibits pyroptosis in synoviocytes from osteoarthritic joints via activation of the AMPK signaling pathway
Source: Inflammopharmacology. 2021 Jun 3;29(3):695–704. doi: 10.1007/s10787-021-00814-x (PMC8233244; doi:10.1007/s10787-021-00814-x)
Supplement: Supplementary file 1 — Supplementary file1 (DOCX 358 kb) [file 10787_2021_814_MOESM1_ESM.docx]

**Supplementary Figure
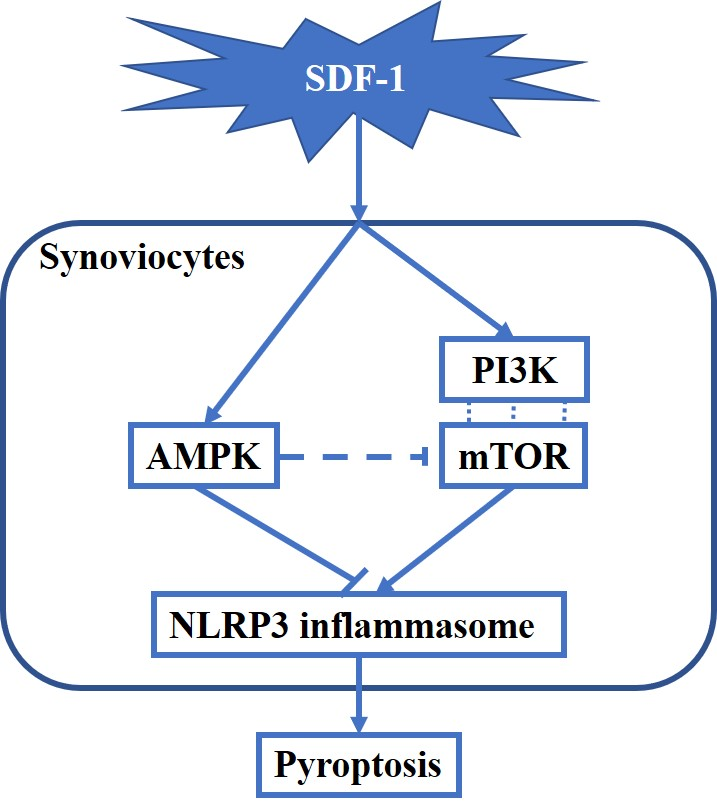
1.** Effects and potential mechanism of SDF-1 on OA FLS NLRP3 inflammasome and pyroptosis.
