# Supplementary material for: Exogenous stromal cell-derived factor-1 (SDF-1) suppresses the NLRP3 inflammasome and inhibits pyroptosis in synoviocytes from osteoarthritic joints via activation of the AMPK signaling pathway
Source: Inflammopharmacology. 2021 Jun 3;29(3):695–704. doi: 10.1007/s10787-021-00814-x (PMC8233244; doi:10.1007/s10787-021-00814-x)
Supplement: Supplementary file 2 — Supplementary file2 (DOCX 13 kb) [file 10787_2021_814_MOESM2_ESM.docx]

Supplementary Table 1. Sequences of primers

| Gene | Forward primer(5`-3`) | Reverse primer |
| --- | --- | --- |
| *NLRP3* | GCTTGCCGACGATGCCTTCC | TGATGACAACAACACCCGATGCTG |
| *ASC* | CTTATCGCGAGGGTCACAAA | AGCTTCCGCATCTTGCTT |
| *Caspase-1* | CTGCTCTTCCACACCAGATAAT | TTTCCTCCACATCACAGGAAC |
| *GSDMD* | TGGTGTTGTCCTCCGGAATG | GCGTTTCACTCAGCATGGTC |
| *IL-1β* | GTGGTGTTCTCCATGTCCTT | CAGCTGTAGAGTGGGCTTATC |
| *SDF-1* | AACTGTGCCCTTCAGATTGT | CAGGTACTCCTGAATCCACTTTAG |
| *GAPDH* | GCACCGTCAAGGCTGAGAAC | TGGTGAAGACGCCAGTGGA |
