# Supplementary material for: Exogenous stromal cell-derived factor-1 (SDF-1) suppresses the NLRP3 inflammasome and inhibits pyroptosis in synoviocytes from osteoarthritic joints via activation of the AMPK signaling pathway
Source: Inflammopharmacology. 2021 Jun 3;29(3):695–704. doi: 10.1007/s10787-021-00814-x (PMC8233244; doi:10.1007/s10787-021-00814-x)

## Slide 1
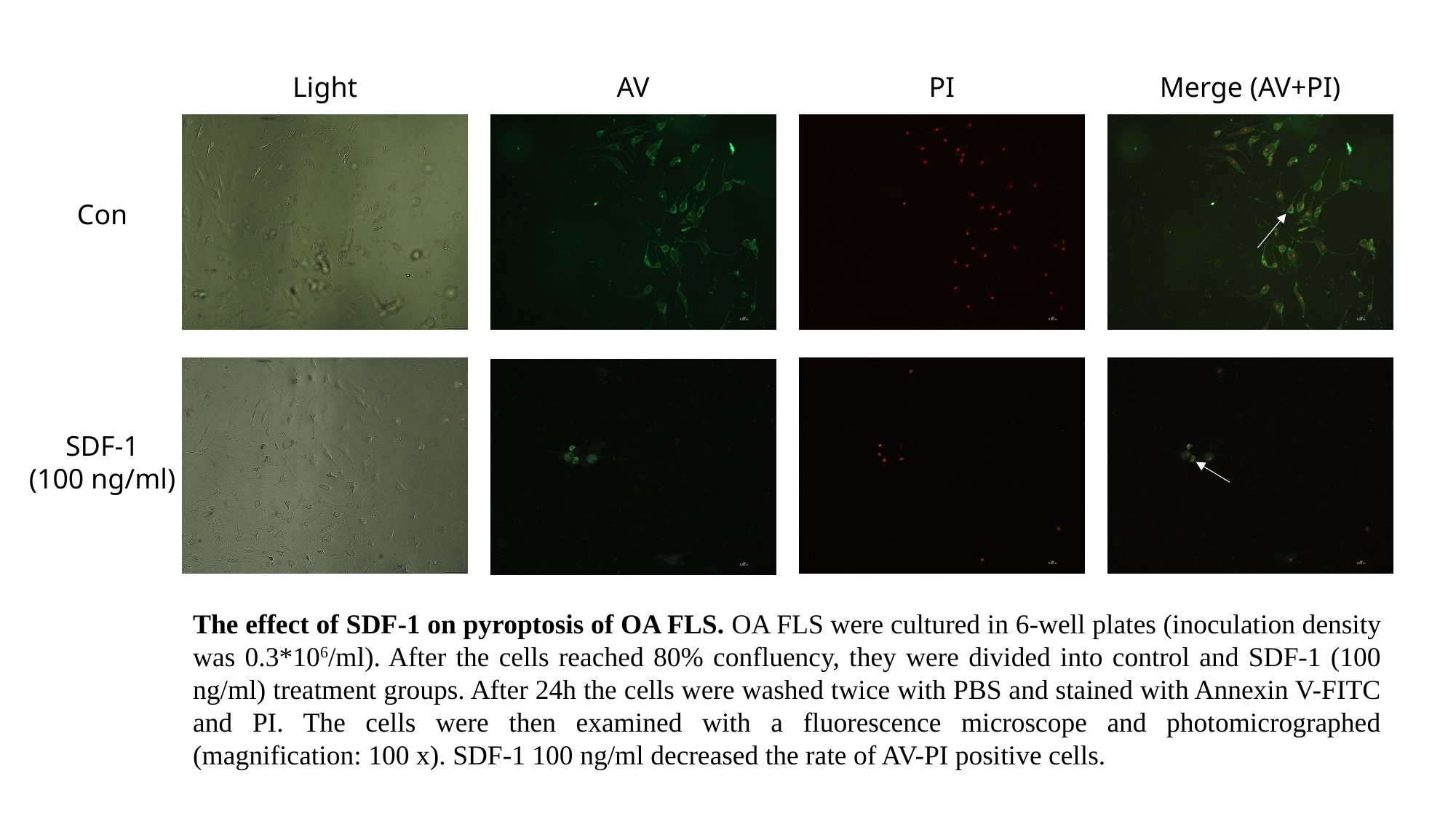

Light
AV
PI
Merge (AV+PI)
Con
SDF-1
(100 ng/ml)
The effect of SDF-1 on pyroptosis of OA FLS. OA FLS were cultured in 6-well plates (inoculation density was 0.3*106/ml). After the cells reached 80% confluency, they were divided into control and SDF-1 (100 ng/ml) treatment groups. After 24h the cells were washed twice with PBS and stained with Annexin V-FITC and PI. The cells were then examined with a fluorescence microscope and photomicrographed (magnification: 100 x). SDF-1 100 ng/ml decreased the rate of AV-PI positive cells.

## Slide 2
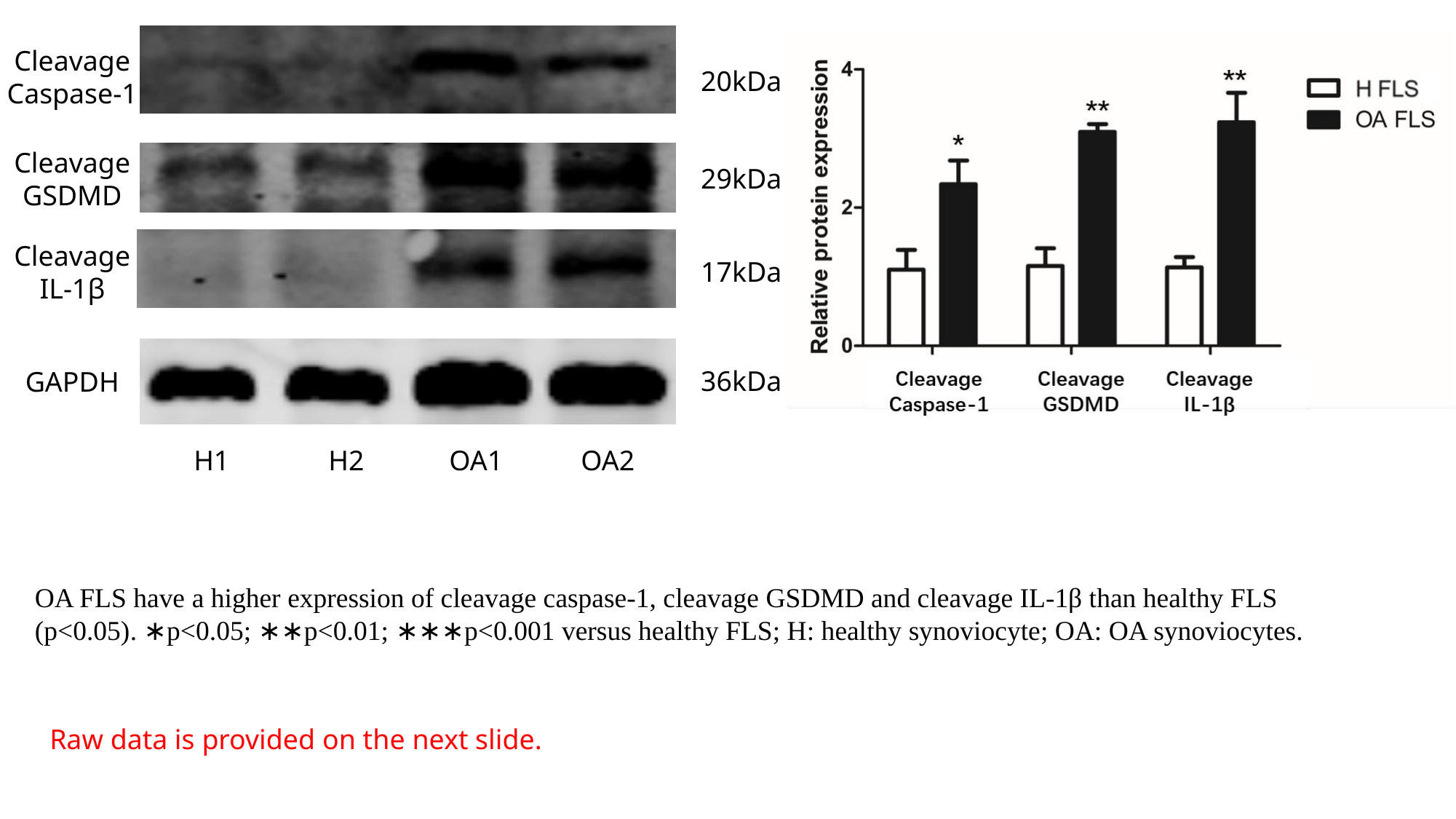

Cleavage
Caspase-1
20kDa
Cleavage
GSDMD
29kDa
Cleavage
IL-1β
17kDa
36kDa
GAPDH
 H1 H2 OA1 OA2
OA FLS have a higher expression of cleavage caspase-1, cleavage GSDMD and cleavage IL-1β than healthy FLS (p<0.05). ∗p<0.05; ∗∗p<0.01; ∗∗∗p<0.001 versus healthy FLS; H: healthy synoviocyte; OA: OA synoviocytes.
Raw data is provided on the next slide.

## Slide 3
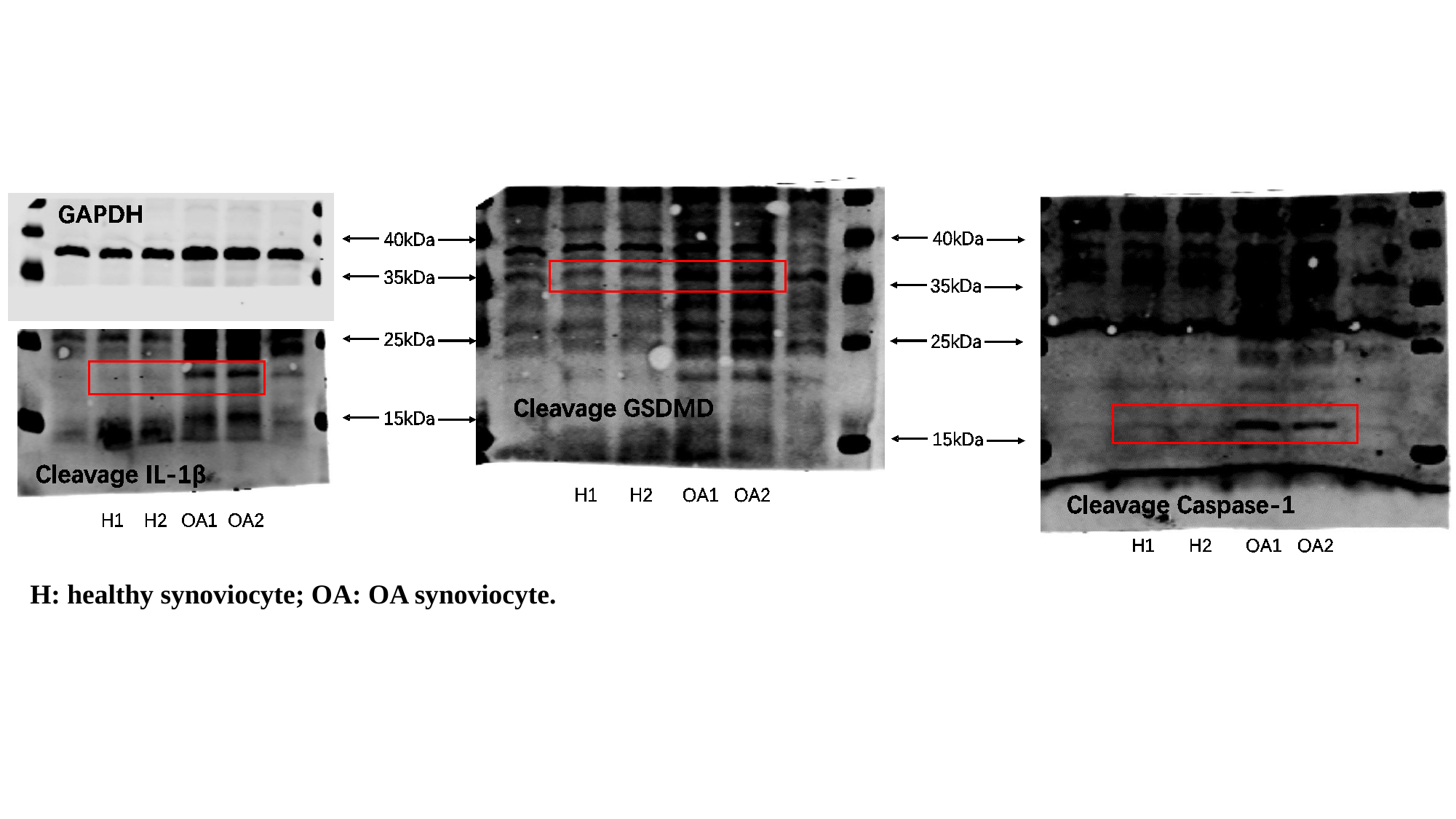

H: healthy synoviocyte; OA: OA synoviocyte.

## Slide 4
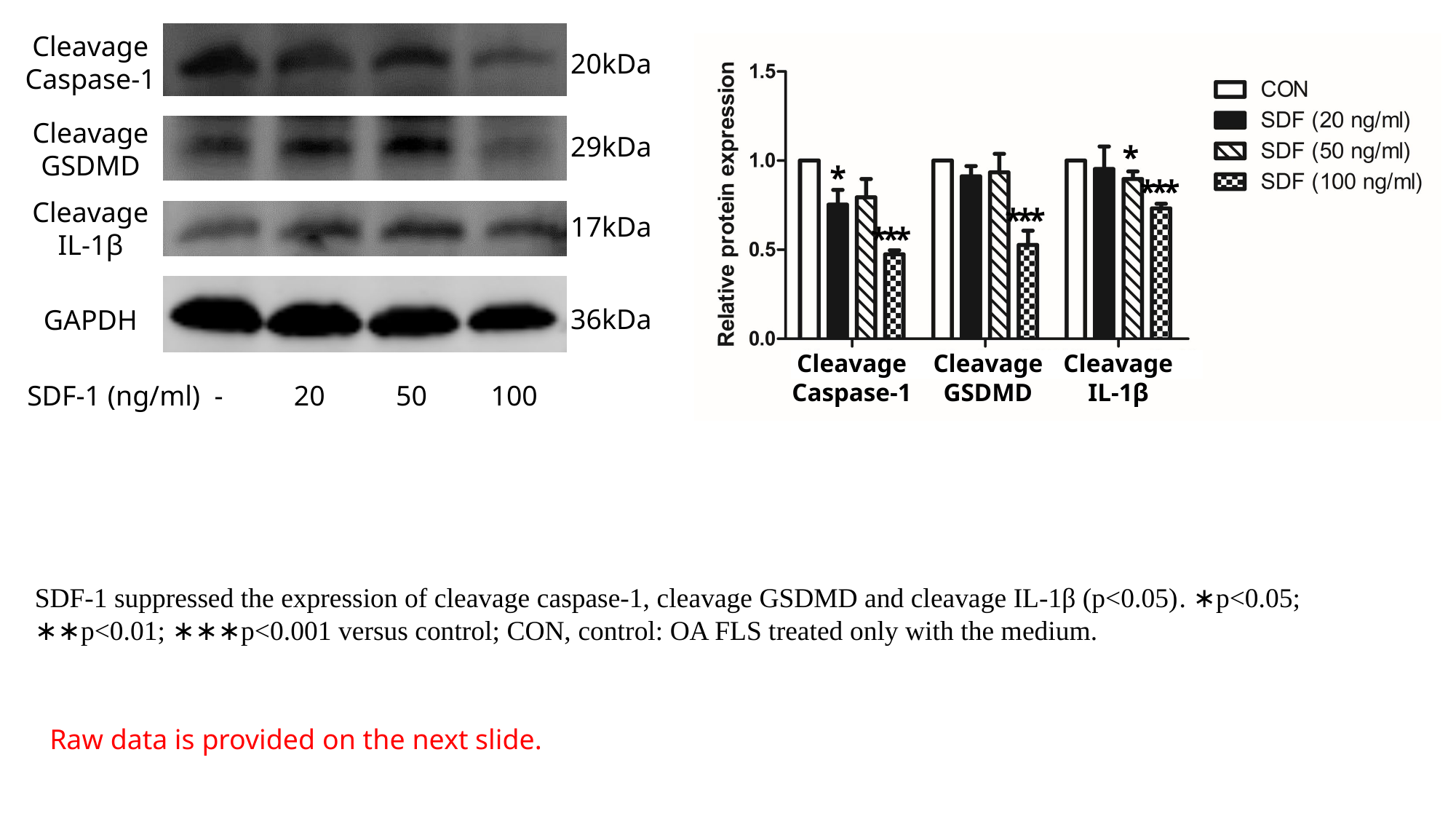

Cleavage
Caspase-1
20kDa
Cleavage
GSDMD
29kDa
Cleavage
IL-1β
17kDa
36kDa
GAPDH
SDF-1 (ng/ml) - 20 50 100
*
*
***
***
***
Cleavage
Caspase-1
Cleavage
GSDMD
Cleavage
IL-1β
SDF-1 suppressed the expression of cleavage caspase-1, cleavage GSDMD and cleavage IL-1β (p<0.05). ∗p<0.05; ∗∗p<0.01; ∗∗∗p<0.001 versus control; CON, control: OA FLS treated only with the medium.
Raw data is provided on the next slide.

## Slide 5
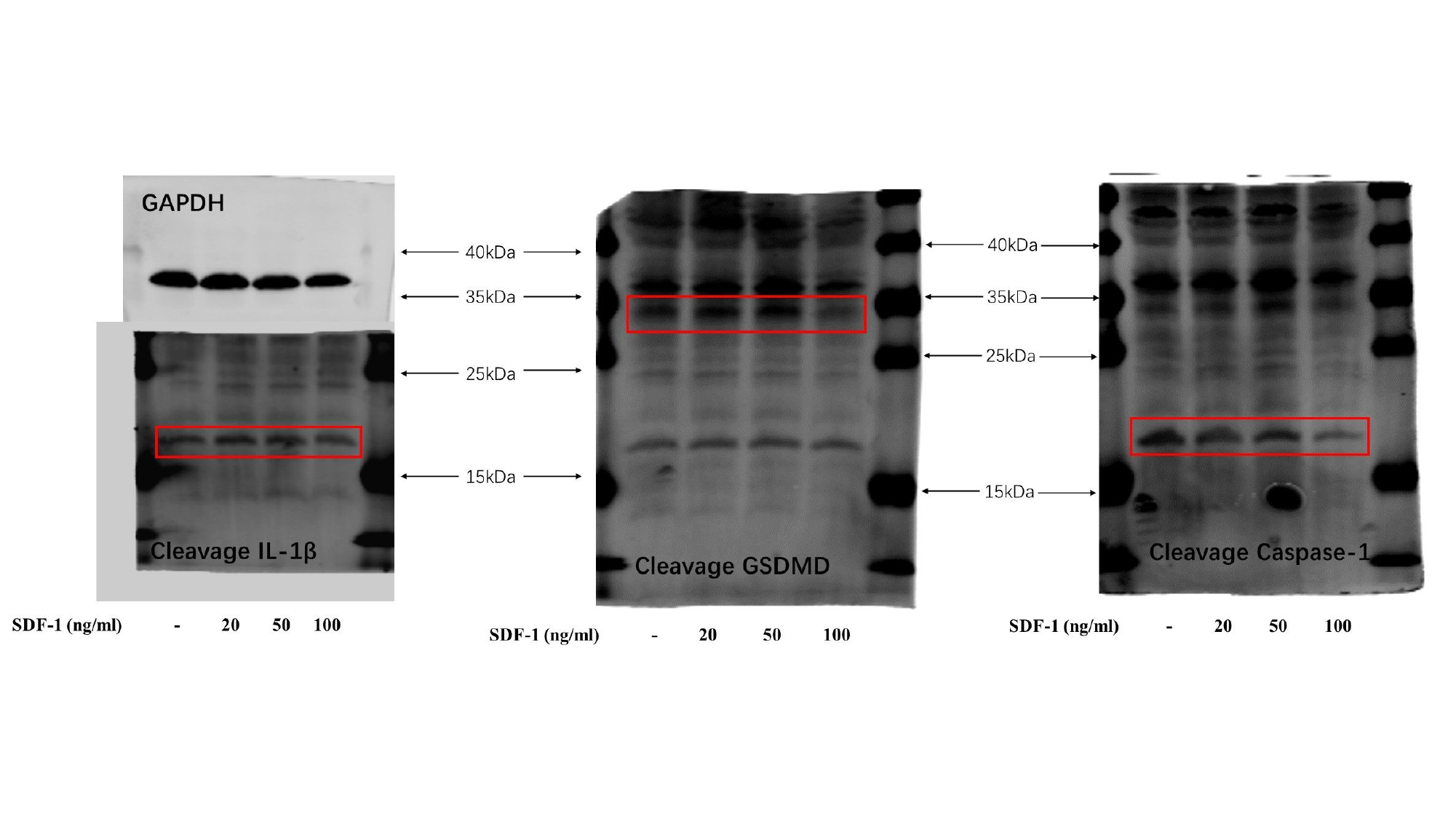

Supplement: Supplementary file 3 — Supplementary file3 (PPTX 19610 kb) [file 10787_2021_814_MOESM3_ESM.pptx]
