# Supplementary material for: Exogenous stromal cell-derived factor-1 (SDF-1) suppresses the NLRP3 inflammasome and inhibits pyroptosis in synoviocytes from osteoarthritic joints via activation of the AMPK signaling pathway
Source: Inflammopharmacology. 2021 Jun 3;29(3):695–704. doi: 10.1007/s10787-021-00814-x (PMC8233244; doi:10.1007/s10787-021-00814-x)
Supplement: Supplementary file 4 — Supplementary file4 (DOCX 14 kb) [file 10787_2021_814_MOESM4_ESM.docx]

| Product | Catalog Number | Manufacturer |
| --- | --- | --- |
| SDF-1 | Cat. # 350-NS-050 | R&D Systems, Shanghai, China |
| Dorsomorphin | Cat. # HY-13418A | MedChemExpress, Shanghai, China |
| 3MA | Cat. # HY-19312 | MedChemExpress, Shanghai, China |
| Anti-NLRP3 antibody | Cat. # ab 214185 | Abcam, Shanghai, China |
| Anti-caspase-1 antibody | Cat. # ab 207802 | Abcam, Shanghai, China |
| Anti-IL-1β antibody | Cat. # ab2105 | Abcam, Shanghai, China |
| Anti-PI3K antibody | Cat. # ab182651 | Abcam, Shanghai, China |
| Anti-ASC antibody | Cat. # sc-514414 | Santa Cruz Biotechnology, Shanghai, China |
| Anti-GSDMD antibody | Cat. # 96458 | Cell Signaling Technology, Shanghai, China |
| Anti-LC3 antibody | Cat. # 3868S | Cell Signaling Technology, Shanghai, China |
| Anti-phospho AMPK antibody | Cat. # ABP0052 | Abbkine, Wuhan, China |
| Anti-GAPDH antibody | Cat. # A01021 | Abbkine, Wuhan, China |
| HRP goat anti-rabbit IgG antibody | Cat. # A21020 | Abbkine, Wuhan, China |
| HRP goat anti-mouse IgG antibody | Cat. # A21010 | Abbkine, Wuhan, China |
| Anti-mtTor antibody | Cat. # BS1844 | Bioworld Technology, Shanghai, China |
| Anti-p62 antibody | Cat. # A11483 | ABclonal, Wuhan, China |
